# Clinical and Microbiological Characteristics of Febrile Neutropenia During Induction Chemotherapy in Adults With Acute Leukemia

**DOI:** 10.1002/cnr2.2129

**Published:** 2024-08-19

**Authors:** Sandra Rajme‐López, Andrea C. Tello‐Mercado, Edgar Ortíz‐Brizuela, Bernardo A. Martínez‐Guerra, Karla M. Tamez‐Torres, Carla M. Román‐Montes, María F. González‐Lara, Alfredo Ponce‐de‐León

**Affiliations:** ^1^ Infectious Diseases Department Instituto Nacional de Ciencias Médicas y Nutrición Salvador Zubirán Mexico City Mexico; ^2^ Internal Medicine Department Instituto Nacional de Ciencias Médicas y Nutrición Salvador Zubirán Mexico City Mexico; ^3^ Clinical Microbiology Laboratory Instituto Nacional de Ciencias Médicas y Nutrición Salvador Zubirán Mexico City Mexico

**Keywords:** acute leukemia, febrile neutropenia, infectious source

## Abstract

**Background:**

Few studies regarding infectious causes of febrile neutropenia (FN) in Mexico are available.

**Aims:**

We aimed to describe clinical and microbiological characteristics of FN episodes during induction chemotherapy in adults with acute leukemia.

**Methods and Results:**

This retrospective cohort from a Mexican tertiary care center included adults with newly diagnosed acute leukemia between January 2014, and December 2018. Clinical and microbiological characteristics were summarized using descriptive statistics. Univariate analyses for associations between clinical characteristics and FN and/or death were made; logistic regression analysis was performed to assess relationships with FN. Kaplan–Meier survival estimates were modeled for antimicrobial prophylaxis and FN. Ninety‐five patients were included. Median age was 28 (IQR 20–43), 49 (52%) were males, and 74 (78%) developed FN (74/95). Among these, 98% had an identified source of infection (73/74) and 65% had >1. Common infections were urinary tract infection (24%), bacterial sinusitis (20%), and bacterial pneumonia (19%). Gram‐negatives were the most frequently isolated microorganisms (69%), followed by Gram‐positives (21%), and fungi (9%). Antimicrobial prophylaxis was inversely associated with FN (aOR = 0.07, CI 0.008–0.060, *p* = 0.02). Invasive fungal diseases were associated with 30‐day mortality (aOR = 9.46, 95% CI 1.66–54.05).

**Conclusion:**

Infections caused 98% of the FN episodes. Gram‐negative bacteria are the most common pathogens.

## 1 Introduction

Infections in patients with chemotherapy‐induced neutropenia have been described since the beginning of these therapies in the mid‐20th century. Significant changes in mortality have been observed, related to the evolution of microbiological epidemiology, technological advances in diagnosis, the development of new antibiotics, and the emergence of antimicrobial resistance [1]. Chemotherapy‐associated febrile neutropenia (FN) occurs in more than 80% of hematologic patients after the first course of treatment [2]. Typically, an infectious cause can be identified in only 20%–30% of cases, with soft tissue, gastrointestinal, pulmonary, and skin infections being the most common causes [2, 3].

The trends of frequency of distinct causal pathogens of FN have changed significantly over time. Currently, Gram‐negative bacilli (GNB) are the most frequently isolated agents in patients with FN [2, 4, 5]. Within this group, the most common pathogens are *E. coli*, *Klebsiella* spp., *Enterobacter* spp., *Pseudomonas aeruginosa*, *Citrobacter* spp., *Acinetobacter* spp., and *S. maltophila* [2, 4, 5, 6]. Based on reported microbiology, international guidelines recommend coverage against *P. aeruginosa* as an essential component of initial empiric treatment [2, 7].

Because of improvements in diagnosis, the development of new antibiotics, and the emergence of antimicrobial resistance, significant changes in mortality rates have also been recently reported [1, 7]. When an agent is isolated or the source of infection is identified, treatment must be tailored accordingly. However, initial empirical treatment is often challenging and depends primarily on local epidemiological data [2, 3, 8].

There are few studies regarding specific infectious causes and causal pathogens in febrile neutropenic patients in Mexico. In a study published in 2000, primary bacteremia was identified as the most common source in hematologic patients with severe neutropenia, followed by infections related to intravascular devices and soft tissue infections. The pathogens isolated were, in decreasing order, *E. coli*, coagulase‐negative staphylococci, and *K. oxytoca* [9]. In another retrospective study conducted in 2006, examining patients with acute myeloid leukemia, a 96% incidence of FN was found. Analysis of isolates in blood cultures found gram‐negative bacteria to be the most common microorganism [10].

International guidelines have not been updated recently, so the recommendations issued may not be suitable for most settings. Knowledge of the current landscape of the infectious causes of FN in specific settings could aid in timely diagnosis and treatment [11]. This study aims to describe the clinical and microbiological characteristics of patients with acute leukemia (AL) who develop FN during induction chemotherapy.

## 2 Methods

This is a retrospective cohort study at Instituto Nacional de Ciencias Médicas y Nutrición “Salvador Zubirán,” a tertiary care center in Mexico City. Adult patients (≥18 years) with newly diagnosed AL who received induction chemotherapy at our institution between January 1, 2014, and December 31, 2018 were included. Clinical and microbiological information was retrieved from electronic medical records. Hematological diagnoses were classified using the International Consensus Classification of Myeloid Neoplasms and Acute Leukemias [12]. Induction chemotherapy was defined as the first chemotherapy regime received after AL diagnosis to reach remission. FN was defined as an oral temperature ≥38.3°C or two consecutive measurements ≥38.0°C for 2 h and an absolute neutrophil count (ANC) <500 cells/mL [8].

Infections attributed to FN episodes were reviewed by an infectious diseases specialist, and classified into 14 categories: primary bacteremia, central line associated bloodstream infection [13], *C. difficile* infection (CDI) [14], herpes simplex, intraabdominal infection [15], neutropenic colitis [16], odontogenic abscess, perianal abscess, pharyngitis, pneumonia [17], proven/probable invasive fungal disease (IFD) [18], sinusitis, skin and soft tissue infection (SSTI) [19], and urinary tract infection (UTI) [20].

Antibiotic resistance for the most common causal bacteria was evaluated by the VITEK2 MS system (ciprofloxacin and ESBL production) or by the broth microdilution method (carbapenems). This was reported as frequencies/proportions.

### 2.1 Statistical Analysis

Clinical and microbiological characteristics were summarized using mean and standard deviation (SD) or median and interquartile range (IQR), as appropriate. Primary outcomes were FN after induction chemotherapy and death after induction chemotherapy. Univariate analysis to identify associations between the different clinical characteristics and FN or death was made using Fisher's exact test or exact *T*‐test. Logistic regression analysis for potential risk factors associated with FN or death was performed. Variables with a *p*‐value <0.2 in the univariate analysis and those with biological plausibility were included in the model. Kaplan–Meier survival analysis was modeled for antimicrobial prophylaxis and the primary outcome. Adjusted odds ratios (aOR) and 95% confidence intervals (CI) were calculated. A two‐sided *p*‐value <0.05 was considered significant. Statistical analysis was done using R version 4.3.1 [21].

## 3 Results

During the study period, 100 patients with newly diagnosed AL attended our institution. Five were excluded: two received chemotherapy at another center, two received treatment for FN in a different center, and one had incomplete data. Ninety‐five patients were included. Median age was 28 years (IQR 20–43), 49/95 were male (52%), and 74/95 developed FN (78%). All patients received chemotherapy through a central venous catheter. The two most common chemotherapy regimes used were HCVAD (51%) and an institutional protocol (25%) [22]. The rest of the baseline clinical characteristics can be found in Table 1.

**TABLE 1:** Baseline clinical characteristics.

	All	No febrile neutropenia	Febrile neutropenia	*p*
	*n* = 95	*n* = 21	*n* = 74	
Age years, median (IQR)	28 (20–43)	28 (20–44)	27 (20–42)	0.90
Male sex, *n* (%)	49 (52)	8 (38)	41 (55)	0.25
Charlson comorbidity index score, median (IQR)	2 (2–2)	2 (2–2)	2 (2–2)	0.43
Diagnosis, *n* (%)				0.03
Acute lymphoblastic leukemia	80 (84)	18 (86)	62 (84)	
Acute myeloblastic leukemia	10 (11)	0	10 (13)	
Acute promyelocytic leukemia	5 (5)	3 (14)	2 (3)	
Chemotherapy, *n* (%)				0.42
HCVAD	48 (51)	8 (38)	40 (54)	
Institutional protocol	24 (25)	7 (33)	17 (23)	
Other	23 (24)	6 (29)	17 (23)	
Steroid, *n* (%)	85 (90)	21 (100)	64 (87)	0.17
Immunomodulating biologic agents^a^, *n* (%)	27 (28)	7 (33)	20 (27)	0.77
Infection before diagnosis, *n* (%)	30 (32)	6 (29)	24 (32)	0.94
Any antimicrobial prophylaxis, *n* (%)	70 (74)	20 (95)	50 (68)	0.02
Ciprofloxacin	42 (44)	14 (67)	28 (38)	0.04
Acyclovir	33 (35)	9 (43)	24 (32)	0.53
Fluconazole	36 (38)	7 (33)	29 (39)	0.82
Itraconazole	31 (33)	11 (52)	20 (27)	0.05
Length of stay days, median (IQR)	35 (28–44)	28 (23–34)	38 (29–50)	<0.001
30‐day mortality, *n* (%)	8 (8)	1 (5)	7 (10)	0.81

Abbreviations: HCVAD = hyper‐CVAD (cyclophosphamide, vincristine, doxorubicin, dexamethasone, methotrexate, and cytarabine), IQR = interquartile range.

^a^ Rituximab or imatinib.

Before starting chemotherapy, 30 patients had an infection, most commonly community acquired pneumonia (CAP) [10], UTI [5], and primary bacteremia [3]. Cultures were positive in 15 of them. NonESBL‐producing *E. coli* [4], methicillin‐sensitive *S. aureus* [2], and susceptible *P. aeruginosa* [2] were the most frequent microorganisms.

**FIGURE 1:**
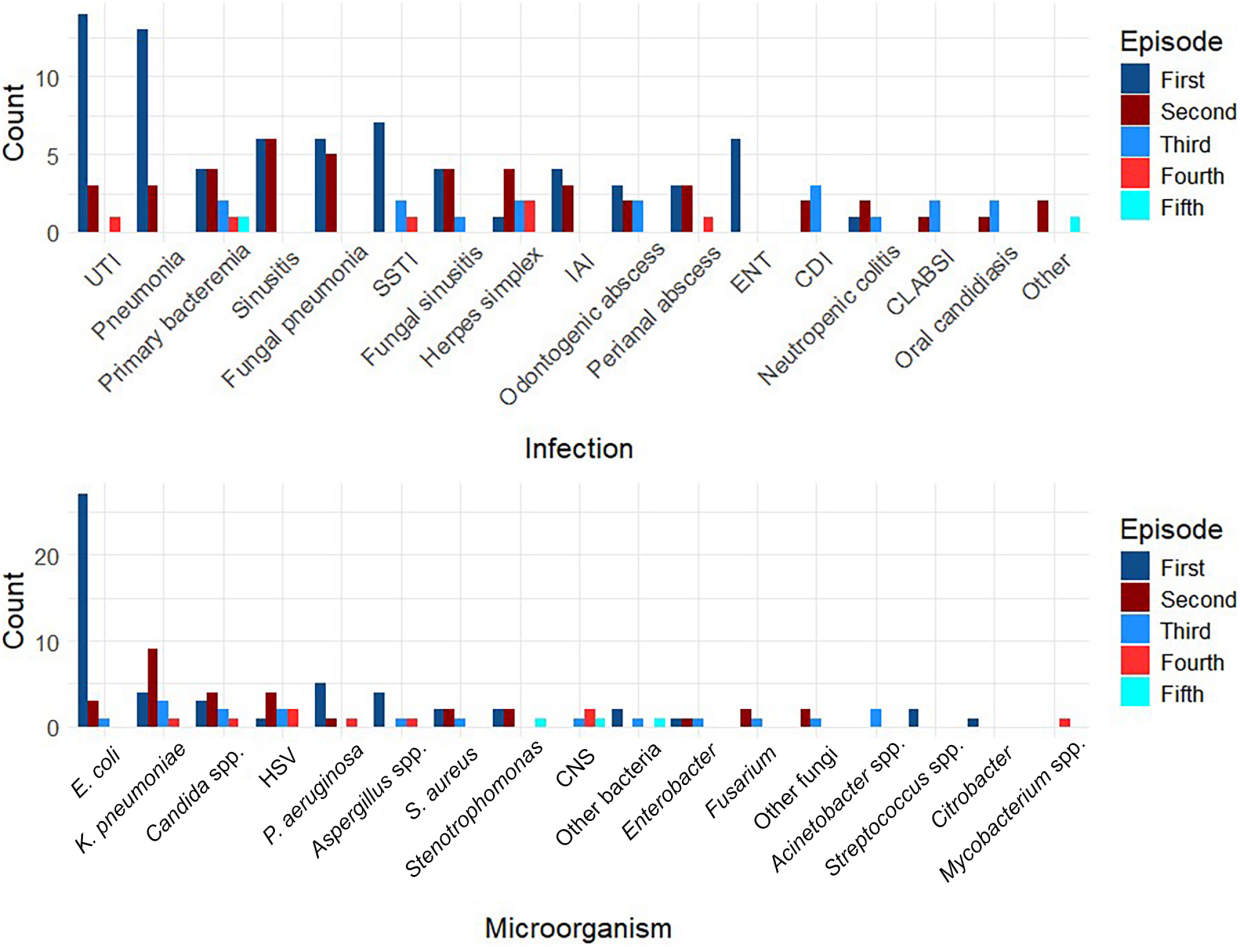
Sequential microbiological etiologies and sources of infection of febrile neutropenia episodes. Top: Sites of infection by infectious episode. Bottom: Causal microorganism by infectious episode.

Among patients who developed FN after chemotherapy, 73/74 had an identified source of infection (98%), and 48/74 had more than one infection (65%). All patients received empirical antimicrobial treatment, with antipseudomonal carbapenems being the most frequent choice (89%). Screening tests included a chest x‐ray and central and peripheral blood cultures for all patients. Additional tests were ordered according to the suspected source of infection. Overall, the most common infection was UTI (24%), followed by bacterial sinusitis (20%), bacterial pneumonia, (19%) and primary bacteremia (16%). Other information on the episodes of FN can be found in Table 2. There were 101 positive microbiological cultures/tests. GNB were the most frequent microorganisms isolated (69%), followed by Gram‐positive bacteria (21%), and fungi (9%). Table 3 shows the most common microorganisms isolated with their corresponding isolation site. Polymerase chain reaction (PCR) confirmed herpes simplex virus infection in 9%. Detailed information on the sequential occurrence of distinct pathogens and infectious sources can be found in Figure 1.

**TABLE 2:** Clinical characteristics of febrile neutropenia episodes.

	All	Alive	Dead	*p*
	*n* = 74	*n* = 67	*n* = 7	
Diagnosis, *n* (%)				0.90
Acute lymphoblastic leukemia	62 (84)	56 (84)	6 (86)	
Acute myeloblastic leukemia	10 (14)	9 (13)	1 (14)	
Acute promyelocytic leukemia	2 (2)	2 (3)	0	
Chemotherapy, *n* (%)				0.83
HCVAD	40 (54)	36 (54)	4 (57)	
Institutional protocol	17 (23)	15 (22)	2 (29)	
Other	17 (23)	16 (24)	1 (14)	
Steroid, *n* (%)	64 (87)	58 (87)	6 (86)	1.00
Immunomodulating biologic agents^a^, *n* (%)	20 (27)	19 (28)	1 (14)	0.73
Any antimicrobial prophylaxis, *n* (%)	50 (68)	45 (67)	5 (71)	1.00
Ciprofloxacin	28 (38)	25 (37)	3 (43)	1.00
Itraconazole	20 (27)	17 (25)	3 (43)	1.00
Fluconazole	29 (39)	26 (39)	3 (43)	1.00
Acyclovir	24 (32)	22 (33)	2 (29)	0.59
Infection before diagnosis, *n* (%)	24 (32)	21 (31)	3 (43)	0.85
ANC, median (IQR)	21 (6–146)	21 (6–123)	56 (9–191)	0.58
SOFA score, median (IQR)	5 (4–6)	5 (4–5)	9 (7–11)	0.008
Invasive mechanical ventilation, *n* (%)	7 (10)	3 (5)	4 (57)	<0.001
Vasopressor, *n* (%)	18 (24)	13 (19)	5 (71)	0.01
Identified infection source, *n* (%)	73 (97)	66 (99)	7 (100)	1.00
UTI	18 (24)	17 (25)	1 (14)	0.85
Bacterial sinusitis	15 (20)	15 (22)	0	0.36
Bacterial pneumonia	14 (19)	13 (19)	1 (14)	1.00
Primary bacteremia	12 (16)	11 (16)	1 (14)	1.00
Fungal pneumonia	9 (12)	9 (12)	1 (14)	1.00
Fungal sinusitis	9 (12)	5 (8)	4 (57)	0.001
SSTI	9 (12)	8 (12)	1 (14)	1.00
Herpes simplex	9 (12)	8 (12)	1 (14)	1.00
Odontogenic abscess	7 (10)	6 (9)	1 (14)	1.00
Perianal abscess	7 (10)	7 (10)	0	0.83
IAI	6 (8)	4 (6)	2 (29)	0.18
Neutropenic colitis	5 (7)	5 (7)	1 (14)	0.97
CDI	5 (7)	4 (6)	1 (14)	0.97
ENT infection	4 (5)	4 (6)	0	1.00
Other	3 (4)	2 (3)	1 (14)	0.98
Fungaemia	3 (4)	2 (3)	1 (14)	0.66
IFD	19 (26)	14 (21)	5 (71)	0.014
>1 source	48 (65)	42 (63)	6 (86)	0.43
Length of stay days, median (IQR)	37 (29–50)	37 (29–48)	39 (26–54)	0.76

Abbreviations: ANC = absolute neutrophil count, CDI = *C. difficile* infection, ENT = ear, nose, throat, HCVAD = hyper‐CVAD (cyclophosphamide, vincristine, doxorubicin, dexamethasone, methotrexate, and cytarabine), IAI = intraabdominal infection, IFD = invasive fungal disease, IQR = interquartile range, SOFA = sespsis related organ failure assessment, SSTI = skin and soft tissue infection, UTI = urinary tract infection.

^a^ Rituximab or imatinib.

**TABLE 3:** Causal microorganisms by culture site.

	Blood	Urine	Respiratory sample	Deep abscess	Biliary tract	Skin/soft tissue
*E. coli*	18	7	0	5	1	0
*K. pneumoniae*	9	3	0	3	1	1
*Enterobacter* spp.	1	2	0	0	0	0
*Citrobacter* spp.	1	0	0	0	0	0
*Proteus* spp.	0	1	0	1	0	0
*P. aeruginosa*	6	0	1	0	0	0
*Acinetobacter* spp.	1	0	1	0	0	0
*Stenotrophomonas* spp.	3	0	2	0	0	
*S. aureus*	2	0	0	2	0	1
*Streptococcus* spp.	2	0	0	0	0	0
*Mycobacterium* spp.	1	0	0	0	0	0
*Coagulase‐negative staphylococcus*	3	0	0	0	0	1
*Candida* spp.	3	2	0	4	1	0
*Aspergillus* spp.	0	0	6	0	0	0
*Fusarium* spp.	0	0	2	0	0	1

Antimicrobial resistance patterns for the three most common microorganisms were the following. Ciprofloxacin resistance: *E. coli* 27/32 (84%), *K. pneumoniae* 4/17 (24%), and *P. aeruginosa* 2/7 (29%). ESBL‐producing: *E. coli* 22/32 (69%) and *K. pneumoniae* 7/17 (41%). Carbapenem resistance: *E. coli* 2/32 (6%), *K. pneumoniae* 2/17 (12%), and *P. aeruginosa* 4/7 (57%).

**FIGURE 2:**
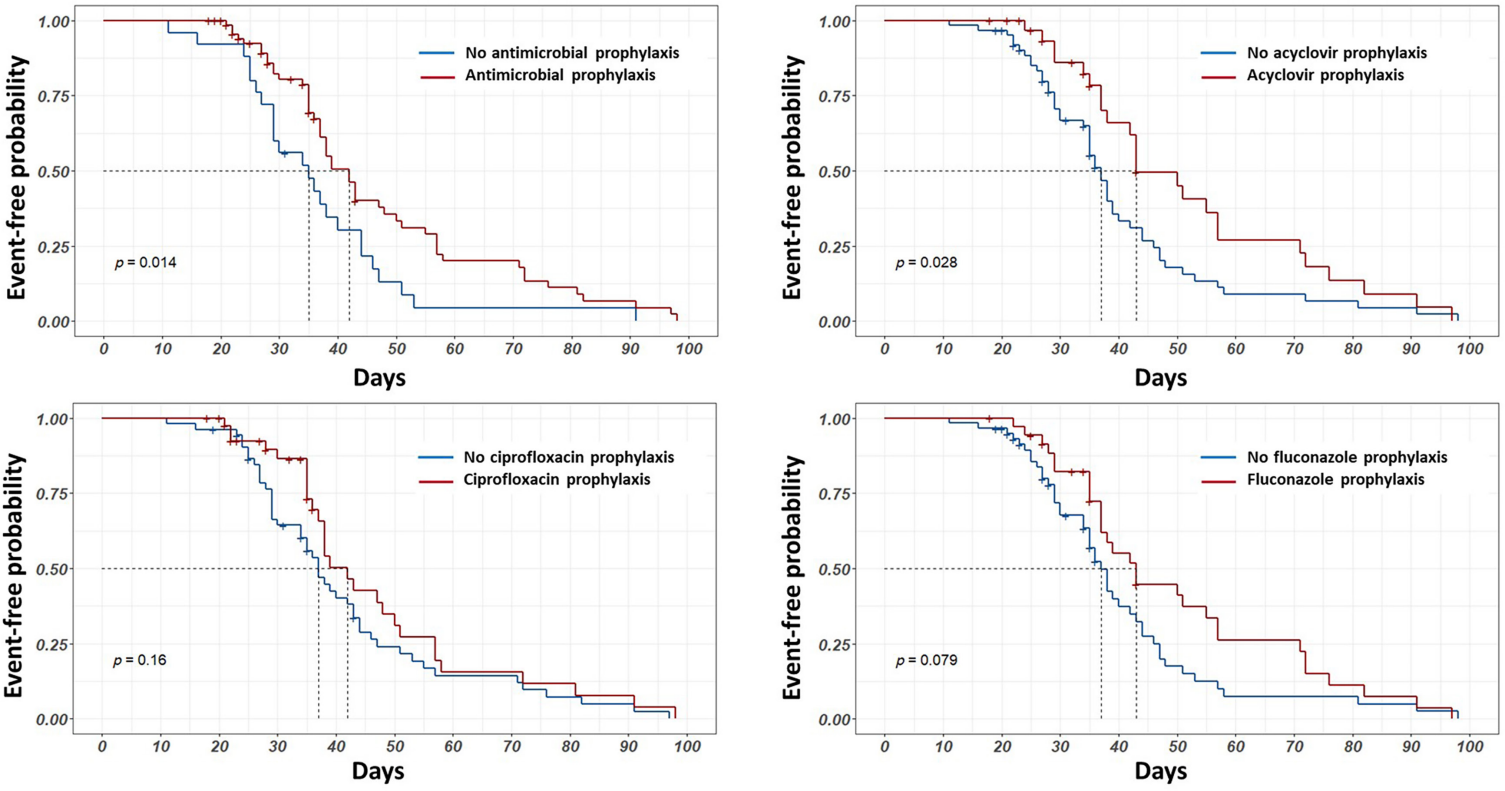
Kaplan–Meier curves for antimicrobial prophylaxis and febrile neutropenia. Top left: Any antimicrobial prophylaxis. Top right: Acyclovir prophylaxis. Bottom left: Ciprofloxacin prophylaxis. Bottom right: Fluconazole prophylaxis.

Any antimicrobial prophylaxis was prescribed in 70/95 (74%) of all patients. The most common agents used were ciprofloxacin 42/95 (44%), acyclovir 33/95 (35%), fluconazole 36/95 (38%), itraconazole 31/95 (33%), or a combination of these. After adjusting for the Charlson comorbidity index score, hematological diagnosis, chemotherapy regime, infection before chemotherapy and steroid or biologic treatments, and logistic regression analysis (Table 4) revealed antimicrobial prophylaxis as the only factor associated with FN, (aOR = 0.07, CI 0.008–0.060, *p* = 0.02). Kaplan–Meier estimates showed different survival effects, according to the antimicrobial prophylaxis received (Figure 2).

**TABLE 4:** Logistic regression analysis of potential risk factors for febrile neutropenia.

	Unadjusted OR	*p*	Adjusted OR	*p*
	(95% CI)		(95% CI)	
Charlson comorbidity index score	1.5 (0.6–3.9)	0.39	2.4 (0.8–7.0)	0.11
Diagnosis				
Acute lymphoblastic leukemia	Ref		Ref	
AML/other	1.2 (0.3–4.6)	0.83	0.23	0.18
Chemotherapy regime				
HCVAD	Ref		Ref	
Other	0.52 (0.2–1.4)	0.20	0.5 (0.2–1.7)	0.29
Steroids	0.1 (0.01–99.9)	1.00	0.1 (0.01–99.9)	1.00
Biologics	0.7 (0.3–2.1)	0.57	0.5 (0.1–1.8)	0.30
Infection before chemotherapy	1.2 (0.4–3.5)	0.74	0.7 (0.2–2.6)	0.63
Antimicrobial prophylaxis	0.1 (0.01–0.8)	0.03	0.07 (0.008–0.6)	0.02

Abbreviations: AML = acute myeloblastic leukemia, HCVAD = hyper‐CVAD (cyclophosphamide, vincristine, doxorubicin, dexamethasone, methotrexate, and cytarabine), OR = odds ratio.

Length of stay was significantly longer among patients who developed FN (38, IQR 29–50 vs. 28, 23–34 days, *p* < 0.001). All‐cause 30‐day mortality occurred in eight patients (10% cumulative incidence). IFD (OR 9.46, 95% CI 1.66–54.05) and fungal sinusitis (OR 16.53, 95% CI 2.87–95.37) were the only infections significantly related to this outcome.

## 4 Discussion

The prevalence of FN in this cohort (74%) is much higher than that reported in other adult populations [2, 8]. Additionally, one or more infections were confirmed in 98% of all the FN episodes, which is also higher than reported in the literature, even for hematological malignancies [23]. The most frequent hematological disease in our study was acute lymphoblastic leukemia (ALL), which is uncommon in adults. Moreover, several studies have shown a worse prognosis in terms of survival for Latin American patients with ALL [24, 25]. The elevated prevalence of FN during induction chemotherapy found in our study may account for some of this overall poorer survival of adults with ALL encountered in developing countries. The reasons for the increased prevalence of infections found in our study have yet to be determined. We hypothesize that heterogeneous antimicrobial prophylaxis, prolonged hospital stay, chemotherapy regimes used, and available diagnostic methods might play a role in this. Also, results from this study contrast with others where no infectious source is found in most FN cases [26, 27]. In a large cohort of 231 patients with FN, Al‐Tawfiq et al. [26] found that 72.5% did not have an identifiable source of infection. This difference with the findings from our study might be due to the exhaustive diagnostic evaluation performed at our center, and to improvements in radiology and microbiology tools over the years. Also, all cases from our study were diagnosed with hematological malignancies, while these diagnoses constituted only 56.6% of Al‐Tawfiq's et al. cohort.

Interestingly, 65% of the population had more than one infection during the study period. Overall, the most common sources of infection were UTI, bacterial pneumonia, sinusitis, and SSTI. However, as seen in Figure 1, the sequential occurrence of infections reflects the profound immunosuppression of patients undergoing induction chemotherapy. Fungal infections were most prevalent as second and third infectious episodes, whereas viral infections occurred predominantly as second, third, or fourth infections.

There seems to be a global tendency to shift from Gram negative to Gram positive microorganisms as causal pathogens in FN [28]. However, Gram‐negative organisms were the most frequently isolated pathogens related to FN in our study. In contrast, Joudeh et al. reported on 150 cancer patients with FN. Gram‐positive organisms were the most frequently found, with *E. faecalis* being the most common [27]. These differences reflect the importance of reporting local epidemiological patterns, to adjust empiric therapy accordingly.

The prevalence of the different microorganisms changed according to the infectious episode. *E. coli* was the most common causal pathogen during the first infection, but during the second and third infections, fungal species emerged as causal pathogens. This distribution of pathogens suggests that community‐acquired microorganisms are the most prevalent cause of the first infections, whereas hospital‐acquired microorganisms are more common during second and later infections. Of note, *P. aeruginosa* was uncommon. Recommendations for antimicrobial empirical treatment in FN emphasize the use of antipseudomonal beta‐lactams [2, 23, 28]. Knowledge on local epidemiology might help tailor empirical treatments, especially in the era of antimicrobial resistance.

Drug resistance patterns in the most common pathogens reveal a high prevalence of quinolone resistance, ESBL, and carbapenem resistance. Of note, 61% of the *E. coli* isolated and 41% of the *K. pneumoniae* isolates displayed an ESBL pattern, limiting the therapeutic options for most cases, seeing that they were among the most frequent causal pathogens. Moreover, resistance to carbapenems was seen in 57% of the *P. aeruginosa* isolates and in 12% of the *K. pneumoniae* isolates. This information should be considered when choosing empirical treatments, especially in the setting of prolonged hospital stays.

IFD were more common than reported by other authors [29], and were significantly associated with 30‐day mortality. Less than 50% of the population received any antifungal prophylaxis, and the choices of antifungal prophylaxis were limited by the time the study took place, due to economic and logistic issues. Patients with ALL are underrepresented in antifungal prophylaxis trials because it is a relatively uncommon malignancy in adults [30]. However, results from this study should raise awareness about the high risk for IFD in adults with ALL and the relevance of timely antifungal prophylaxis in this population.

Community‐acquired infections before the diagnosis and onset of chemotherapy were common (30%). The most common causal pathogen of these infections was nonESBL *E. coli*. Of note, there were two infections caused by *P. aeruginosa*, reflecting the profound immunosuppression these patients experience even before initiating chemotherapy. Carbapenem‐sparing antimicrobial regimes for patients with community‐acquired infections seem a reasonable choice, especially when hemodynamically stable and without other risk factors.

Ciprofloxacin was the most common antibacterial agent used for prophylaxis. It is usual in settings like ours to choose this antibiotic due to its low cost and ease of administration [31]. There was a slight tendency for the patients who received ciprofloxacin prophylaxis to be less likely to develop FN, although this finding did not reach statistical significance. However, looking at combined antimicrobial prophylaxis, there was an overall positive effect in preventing FN. The major drivers of this effect were antiviral (acyclovir) and antifungal (fluconazole) prophylaxis, as seen in Figure 2. These findings are inconclusive and warrant a clinical trial to define the best antimicrobial prophylaxis, and to address the controversies of the benefits of fluoroquinolone prophylaxis in the era of antimicrobial resistance [32].

Limitations to this study include the retrospective nature of the design, the diverse antimicrobial prophylaxis regimes used, and the lack of antibiotic consumption data analysis. Strengths of this study include the various chemotherapy regimes reported, a large number of adults with ALL, description of sequential pathogens and infectious syndromes, and use of ciprofloxacin instead of levofloxacin as prophylaxis.

## 5 Conclusion

Infections are the most common cause of FN during induction chemotherapy for hematological malignancies, especially acute lymphoblastic leukemia. Empirical regimes should focus on covering Gram‐negative bacteria. Invasive fungal infections must be suspected and treated promptly. Prospective multicenter studies are needed.

## Author Contributions

Conceptualization: S.R.‐L., M.F.G.‐L., E.O.‐B., and A.P.‐d.‐L. Methodology: S.R.‐L. and E.O.‐B. Formal analysis: S.R.‐L. and E.O.‐B. Investigation: A.C.T.‐M., K.M.T.‐T., and C.M.R.‐M. Resources: A.P.‐d.‐L. Data curation: S.R.‐L., A.C.T.‐M., K.M.T.‐T., and C.M.R.‐M. Writing – original draft preparation: S.R.‐L. and A.C.T.‐M. Writing – review and editing: B.A.M.‐G. and M.F.G.‐L. Visualization: S.R.‐L. Supervision: M.F.G.‐L. and A.P.‐d.‐L. Project administration: S.R.‐L. and E.O.‐B. Funding acquisition: A.P.‐d.‐L.

## Ethics Statement

This work was reviewed and approved by the Instituto Nacional de Ciencias Médicas y Nutrición Salvador Zubirán's Research and Ethics in Research Committees (approval number 4756).

## Consent

Informed consent was waived by the institutional review board due to the retrospective nature of the study.

## Conflicts of Interest

The authors declare no conflicts of interest.

## Data Availability

The data that support the findings of this study are available from the corresponding author upon reasonable request.
